# Phase I dose escalation study of 12b80 (hydroxybisphosphonate linked doxorubicin) in naturally occurring osteosarcoma

**DOI:** 10.18632/oncotarget.27801

**Published:** 2020-11-17

**Authors:** Pierre Boyé, Emmanuelle David, François Serres, Quentin Pascal, Franck Floch, Kévyn Geeraert, Virginie Coste, Laurent Marescaux, Sébastien Cagnol, Jean-Yves Goujon, Maxim Egorov, Ronan Le Bot, Dominique Tierny

**Affiliations:** ^1^Oncovet Clinical Research (OCR), Parc Eurasanté, Loos, France; ^2^Oncovet, Villeneuve d’Ascq, France; ^3^Atlanthera, Saint Herblain, France; ^4^Department of Small Animal Teaching Hospital, The Royal (Dick) School of Veterinary Studies and The Roslin Institute, University of Edinburgh, Easter Bush, Edinburgh, UK

**Keywords:** bisphosphonate, bone targeting, canine, doxorubicin, osteosarcoma

## Abstract

Purpose: 12b80 combines doxorubicin bound to a bone targeting hydroxybisphosphonate vector using a pH-sensitive linker, designed to specifically trigger doxorubicin release in an acidic bone tumor microenvironment. This phase I study aimed to determine the safety and toxicity profiles of 12b80 in dogs with naturally occurring osteosarcoma, with the objective to translate findings from dogs to humans.

Experimental Design: Ten client-owned dogs with osteosarcoma were enrolled in an accelerated dose-titration design followed by 3 + 3 design. Dogs received three cycles of 12b80 intravenous injection at 4 mg/kg (*n* = 1), 6 mg/kg (*n* = 2), 8 mg/kg (*n* = 3), and 10 mg/kg (*n* = 4). Endpoints included safety, tolerability, maximum tolerated dose (MTD), and dose-limiting toxicity (DLT).

Results: The MTD of 12b80 was 8 mg/kg (i.e., equivalent dose of doxorubicin of 110 mg/m^2^, range: 93–126). Most adverse events included grade ≤ 2 gastrointestinal disorders and hypersensitivity reactions. No hematological or cardiac DLT were observed at any dose tested.

Conclusions: In dogs, 12b80 is overall well tolerated and expends the MTD of doxorubicin up to four times the standard dose of 30 mg/m^2^. These results demonstrate the potential therapeutic benefit of 12b80 in canine and human osteosarcoma.

## INTRODUCTION

Research in comparative oncology has demonstrated that naturally occurring canine cancers are of valuable and translatable interest for the understanding of human cancer biology and characterization of new therapies [1–4]. Osteosarcoma is the most common primary bone tumor reported in both human and companion dog populations [5, 6]. Canine osteosarcoma is a suitable model for human osteosarcoma due to their similar clinical presentation, molecular features and therapeutic responses, offering a unique opportunity for preclinical modelling to allow development of innovative therapies for human patients [7–11]. In dogs, the prognosis remains poor. The median disease-free interval in dogs with appendicular osteosarcoma following surgery and adjuvant chemotherapy ranges from 135 to 425 days with a median overall survival ranging from 258 to 479 days [12–19]. Therefore, identifying novel strategies targeting cancer cells and more effective treatments are urgently needed to improve survival in both humans and dogs with osteosarcoma.

The most commonly studied adjuvant chemotherapy drugs used in dogs are cisplatin, carboplatin and doxorubicin, administered either as single agents or in concurrent or alternating combinations [12–19]. Despite the numerous chemotherapy protocols described for canine osteosarcoma, there is currently no consensus on the optimal chemotherapy protocol in dogs. For non-operable or metastatic disease, palliative treatments include radiation therapy, chemotherapy, bisphosphonate or oral analgesics, providing short-term pain control ranging from a median of 53 to 202 days [20–24].

Osteosarcoma originates from osteoblasts and promotes tumor osteoid production and tumoral heterotopic bone tissue formation. In addition, concomitant activation of osteoclasts by tumor cells leads to osteolysis and an acidic tumor microenvironment. The molecule 12b80 is a new antineoplastic compound, combining doxorubicin bound to a bone targeting hydroxybisphosphonate (HBP) vector using a pH-sensitive linker [25]. This HBP linked doxorubicin compound was designed to specifically trigger doxorubicin release in an acidic bone tumor microenvironment, promoting the selective uptake of the drug by tumor cells and in turn, decreasing systemic distribution and associated toxicity. In *in vitro* study, 12b80 displays a rapid and sustained targeting of bone tissue [25]. In *in vivo* study, 12b80 allows higher doxorubicin concentration in the tumor bone environment compared to nonvectorized doxorubicin, exhibits strong antitumor effects on rodent orthotopic osteosarcoma, and demonstrates a dose-response therapeutic activity more potent than doxorubicin/zoledronate combination [25].

Based on these preclinical data, the following study was designed to evaluate the safety and tolerability, maximum tolerated dose (MTD) and dose-limiting toxicity (DLT) of 12b80 in companion dogs with naturally occurring osteosarcoma. Preliminary antitumor efficacy of 12b80 was also evaluated. This dose escalation study represents a pilot translational research study for future phase II randomized double-blind trial of 12b80 in naturally occurring osteosarcoma in dogs. In this capacity, companion dogs can serve as valuable corroborative models for assessing the efficacy of novel therapies, some of which could potentially be of therapeutic value in human osteosarcoma.

## RESULTS

### Clinical characteristics and drug dosing

Ten client-owned dogs were enrolled in the clinical trial between September 23, 2015 and October 13, 2016. The last day of follow-up was May 19, 2018. Baseline characteristics are summarized in Table 1.

**Table 1:** Baseline characteristics of dogs treated with 12b80 (*n* = 10)

Baseline characteristics	Dogs treated with 12b80
**Age, years, mean ± SD (range)**	8.3 ± 2.2 (5.6–12.5)
**Body weight, kg, mean ± SD (range)**	44.8 ± 16 (27.7–70.0)
**Sex, no. (%)**	
Male	4 (40%)
Female	6 (60%)
**Breed, no.**	
Rottweiler	2
Dogue de Bordeaux	1
German Shepherd	1
Golden Retriever	1
Great Dane	1
Great Pyrenees	1
Saint Bernard	1
Cross Breed	2
**Tumor location, no. (%)**	
Proximal humerus	4 (40%)
Distal radius	2 (20%)
Distal ulna	2 (20%)
Distal tibia	1 (10%)
Proximal tibia	1 (10%)
**Histopathological subtype, no. (%)**	
Osteoblastic	9 (90%)
Fibroblastic	1 (10%)
**Pretreatment alkaline phosphatase activity (ALP), no. (%)**	
Normal	6 (60%)
Elevated	4 (40%)
**Metastatic disease at the time of enrollment, no. (%)**	
Absent	6 (60%)
Present	4 (40%)

All ten dogs had biopsy-confirmed diagnosis of osteosarcoma. Nine (90%) dogs were diagnosed with an osteoblastic osteosarcoma and one (10%) dog diagnosed with a fibroblastic osteosarcoma. Tumor locations included proximal humerus (*n* = 4), distal radius (*n* = 2), distal ulna (*n* = 2), distal tibia (*n* = 1) and proximal tibia (*n* = 1). At the time of enrollment, macroscopic metastatic disease was identified in four (40%) dogs. Three dogs presented with pulmonary metastatic disease, and one dog with pulmonary and vertebral metastatic disease based on contrast-enhanced computed tomography.

All dogs involved in the trial followed the same protocol over a period of nine weeks, consisting of three cycles of 12b80 intravenous (IV) injections, administered every three weeks (day 1, day 22, day 43). The dose of 12b80 was escalated from 4 mg/kg to 10 mg/kg (Table 2). Dogs started 12b80 treatment at a dose of 4 mg/kg (*n* = 1) and 6 mg/kg (*n* = 2), divided in two intravenous administrations of a 1-hour constant rate infusion over 24 hours. No DLT was observed and additional dogs were enrolled to determine the MTD in a 3 + 3 dose escalation design. Three dogs were treated at a dose of 8 mg/kg and four dogs at a dose of 10 mg/kg. One dog, initially treated with three cycles at 6 mg/kg, had undergone an intra-patient dose escalation to receive 8 mg/kg. In total four dogs were treated at 8 mg/kg. Four dogs developed grade 1 immediate hypersensitivity reactions (IHR) during drug administration including facial pruritus, head shaking, or facial edema: two of these dogs were treated with 12b80 at 8 mg/kg and two dogs were treated at 10 mg/kg. All signs of IHR resolved after a reduction in the rate of the infusion. Therefore, at doses of 8 mg/kg and 10 mg/kg, the administration schedule was changed from two to three (*n* = 3) or four (*n* = 2) intravenous infusions of 1-hour of each over 48 hours.

**Table 2:** Dose escalation design of 12b80 evaluated in ten client-owned dogs with naturally occurring osteosarcoma

Cohort (Number of dogs)	Dose of 12b80/Cycle	Number of cycle administered / dog Mean (range)	Cumulative dose of 12b80 administered Mean (range)	Equivalent dose of doxorubicin / Cycle Mean (range)	Equivalent cumulative dose of doxorubicin administered Mean (range)
Cohort 1 (*n* = 1)	4 mg/kg	*n* = 1	4 mg/kg	48 mg/m^2^	48 mg/m^2^
Cohort 2 (*n* = 2)	6 mg/kg	*n* = 3	18 mg/kg	78 mg/m^2^ (75–81)	234 mg/m^2^ (225–243)
Cohort 3 (*n* = 3)	8 mg/kg	*n* = 2.33 (2–3)	18.7 mg/kg	110 mg/m^2^ (93–126)	257 mg/m^2^ (186–333)
Cohort 4 (*n* = 4)	10 mg/kg	*n* = 2.75 (2–3)	27.5 mg/kg (20–30)	141.8 mg/m^2^ (120–159)	391.5 mg/m^2^ (270–477)

Four doses were evaluated in an accelerated dose-titration design followed by a 3 + 3 design. The dose of 12b80 was escalated from 4 mg/kg to 10 mg/kg. The equivalent calculated dose of doxorubicin administered is shown.

Six (60%) dogs completed the three cycles protocol, including two (2/2, 100%) dogs treated with 6 mg/kg, one (1/3, 33%) dog treated with 8 mg/kg and three (3/4, 75%) dogs treated with 10 mg/kg. Based on the weight of dogs included in each cohort, a 12b80 dose of 4 mg/kg, 6 mg/kg, 8 mg/kg and 10 mg/kg represents, in average, the equivalent calculated dose of doxorubicin of 48 mg/m^2^, 78 mg/m^2^, 110 mg/m^2^, and 141.8 mg/m^2^ respectively. The range of the equivalent cumulative dose of doxorubicin administered for each cohort treated with 12b80 is shown in Table 2. The dog that had an intra-patient dose escalation of 12b80 completed the three cycles at 6 mg/kg and received three additional cycles at 8 mg/kg. This dog received a 12b80 cumulative dose of 42 mg/kg and an equivalent cumulative dose of doxorubicin of 528 mg/m^2^.

### Safety

All ten dogs were evaluable for safety analysis and DLT analysis. Twenty-eight cycles of 12b80 were evaluated and the type, frequency and severity of treatment-emergent adverse events (TEAE) were graded according to the Veterinary Cooperative Oncology Group criteria for adverse events (VCOG-CTCAE), version 1.1 [26]. Thirty-one TEAE were reported in eight (80%) dogs (Table 3). The most frequently reported TEAE included IHR (6/31, 19%), vomiting (6/31, 19%), thrombocytopenia (5/31, 16%), diarrhea (3/31, 10%), anemia (3/31, 10%), neutropenia (2/31, 6%), decreased appetite (2/31, 6%), elevated alanine transaminase (1/31, 3%) and urea (1/31, 3%). The majority (29/31, 94%) of the TEAE were grade 1 or 2. All hypersensitivity reactions, neutropenia and anemia episodes were grade 1. Three thrombocytopenic episodes were grade 1 and two were grade 2. Full hematological recovery was confirmed before initiation of the next 12b80 cycle in all dogs. No grade ≥ 3 hematological or biochemical toxicity were reported.

**Table 3:** Hematological and non-hematological adverse events graded according to the Veterinary Cooperative Oncology Group criteria for adverse events (VCOG-CTCAE), version 1.1

TEAE	4 mg/kg	6 mg/kg	8 mg/kg	10 mg/kg	Overall
**Hematological adverse events, grade (number of TEAE)**					
Anemia	Grade 1 (1)	Grade 1 (1)	—	Grade 1 (1)	Grade 1 (3)
Neutropenia	—	—	Grade 1 (1)	Grade 1 (1)	Grade 1 (2)
Thrombocytopenia	—	Grade 2 (1)	Grade 1 (1)	Grade 1 (2)	Grade 1 (3)
				Grade 2 (1)	Grade 2 (2)
**Non-hematological adverse events, grade (number of TEAE)**					
Vomiting	Grade 1 (1)	—	Grade 1 (1)	Grade 1 (2)	Grade 1 (4)
			Grade 2 (2)		Grade 2 (2)
Diarrhea	—	—	Grade 1 (1)	Grade 1 (1)	Grade 1 (2)
			Grade 2 (1)		Grade 2 (1)
Hypersensitivity reaction	—	—	Grade 1 (2)	Grade 1 (4)	Grade 1 (6)
Decreased appetite	—	—	Grade 1 (1)	Grade 1 (1)	Grade 1 (2)
ALT elevation	—	—	Grade 2 (1)	—	Grade 2 (1)
Urea elevation	—	—	Grade 2 (1)	—	Grade 2 (1)
Phlebitis and necrosis at the IV injection site	—	—	—	Grade 3 (1)	Grade 3 (1)
Septic shock	—	—	—	Grade 5 (1)	Grade 5 (1)

ALT: alanine transaminase; IV: intravenous; TEAE: treatment-emergent adverse events.

Two severe TEAE were observed in two dogs treated with 10 mg/kg and were considered as DLT. One dog developed one grade 3 phlebitis and necrosis at the IV injection site, which required a surgical intervention. This dog had received the 12b80 IV through an indwelling catheter and this event occurred 7 days after the last administration. One dog developed one grade 5 septic shock with septic peritonitis secondary to duodenal perforation. This dog had received concomitant long-term nonsteroidal anti-inflammatory drugs (NSAID) and this event occurred 14 days after the last dose of 12b80.

No DLT occurred at dose of 4 mg/kg, 6 mg/kg and 8 mg/kg and two dogs developed DLT at dose 10 mg/kg. The MTD and the recommended phase I dose were therefore determined at 8 mg/kg.

### Circulating blood cell counts in dogs treated with 12b80

**Figure 1:**
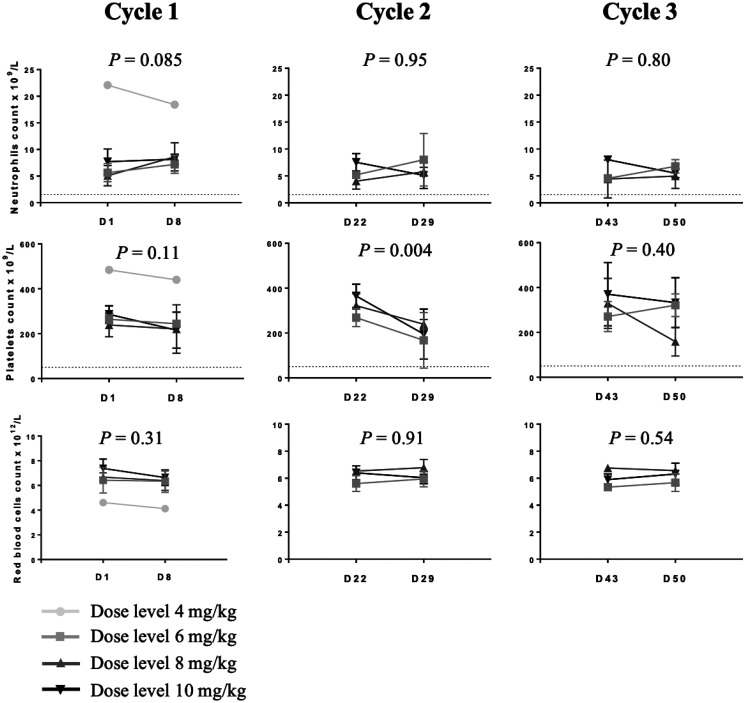
Circulating blood cell counts in dogs treated with 12b80. The protocol included three cycles of 12b80 intravenous injections, administered every three weeks (D1, D22, D43). Neutrophil count of 1.500 × 10^9^/L and platelet count of 75 × 10^9^/L are shown (dotted lines). Data are represented as mean ± SD.

The circulating blood cell counts including neutrophils, platelets and red blood cells before and seven days after each cycle of 12b80 are shown in Figure 1. There was no dose-effect relationship between the dose of 12b80 and the number of neutrophils observed. There was no statistically significant decrease in neutrophil count seven days post treatment. Transient decrease in platelet count occurred 7 days post 12b80 administration. No hematological DLT were observed at any dose tested.

### Cardiac evaluation

**Figure 2:**
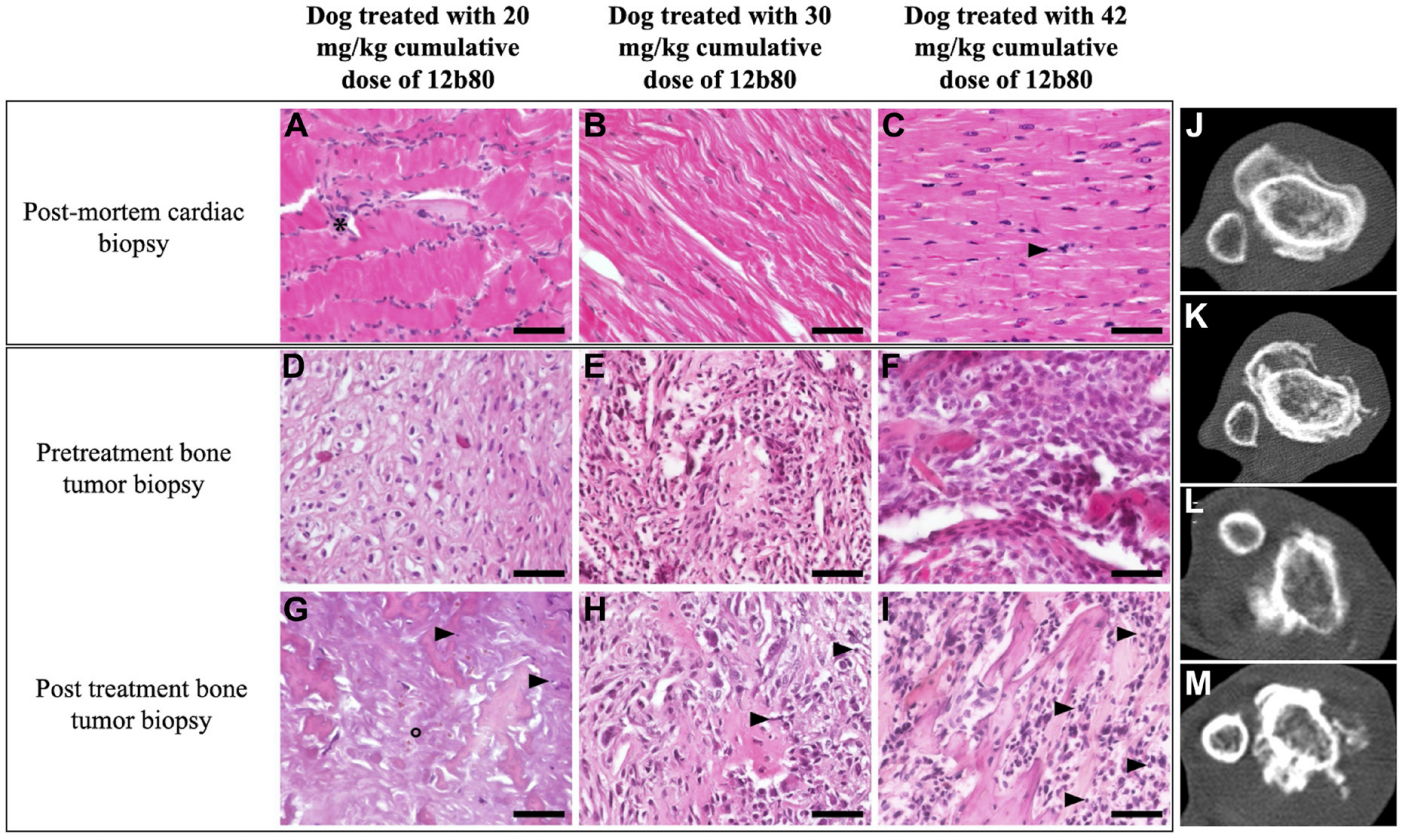
12b80 cardiac toxicity and antitumor activity in dogs. (**A**–**C**) Microscopic photographs of post-mortem cardiac histopathology from three dogs treated with 12b80 cumulative dose of 20 mg/kg (A), 30 mg/kg (B), and 42 mg/kg (C). No dose-limiting cardiotoxicity was noted, rare individual cell necrosis was observed in the dog treated with 42 mg/kg (C, arrowhead), diffuse increased cardiomyocyte cell density was noted in the dog treated with 20 mg/kg (A, ^*^) and no histopathological abnormalities were found in the dog treated with 30 mg/kg (B). Original magnification ×40. Hematoxylin-Eosin staining. Bar = 50 μm. (D–I) Microscopic photographs of bone tumor biopsy before treatment (**D**–**F**) and post treatment (**G**–**I**) in the same three dogs treated with 12b80 cumulative dose of 20 mg/kg (D and G), 30 mg/kg (E and H), and 42 mg/kg (F and I). After treatment, the tumor biopsy shows large necrotic areas with nucleus debris (G–I, arrowheads) or “ghost cells” (G, °). Original magnification ×40. Hematoxylin-Eosin staining. Bar = 50 μm. (**J**–**M**) Bone tumor computed tomography in two dogs treated with 12b80 cumulative dose of 24 mg/kg (J, pretreatment; K, post treatment), and 30 mg/kg (L, pretreatment; M, post treatment). Stable disease was noted at day 57 based on the Response Evaluation Criteria in Solid Tumors version 1.0.

Six (60%) dogs completed the protocol consistent of three cycles of 12b80 and had a follow-up echocardiography performed at day 57. No significant sustained changes in cardiac parameters or cardiac function were identified in any dog. Among these six dogs, three (3/6, 50%) had post-mortem cardiac histopathology performed (Figure 2A–2C). Those dogs received a 12b80 cumulative dose of 20 mg/kg, 30 mg/kg and 42 mg/kg respectively. The first dog received two cycles at 10 mg/kg, the second dog received three cycles at 10 mg/kg and the latter dog was treated with three cycles of 12b80 at 6 mg/kg and three additional cycles at 8 mg/kg. No severe macroscopic or histopathological lesions were observed. A diffuse increase in cardiomyocytes density was reported in the dog treated at 20 mg/kg cumulative dose (Figure 2A) and rare individual cell necrosis was noted in the dog treated at 42 mg/kg cumulative dose (Figure 2C). These histopathological changes were not accompanied by deteriorating cardiac function.

### Efficacy endpoints

Six (60%) dogs completed the three cycles of 12b80 and had a follow-up computed tomography performed at day 57. The response to treatment was evaluated according to the Response Evaluation Criteria in Solid Tumors (RECIST), version 1.0 [27]. Four dogs did not have repeat imaging because of euthanasia for poor quality of life (*n* = 1), grade 5 septic shock (*n* = 1), and exclusion from the trial due to a pathological fracture (*n* = 2). Stable disease was reported in two (2/6, 33%) dogs (Figure 2J–2M). One dog was treated with three cycles of 12b80 at 8 mg/kg and one dog was treated with three cycles of 12b80 at 10 mg/kg. The latter dog presented a persistent stable disease on follow-up computed tomography at four- and six-months post treatment. Four dogs had progressive disease in both primary and metastatic sites at day 57. Subjective pain control was reported by owners in four out of six (67%) dogs after the completion of three cycles of 12b80. The median survival time for all dogs was 57 days (range: 16–766). The median survival time for dogs that completed the three cycles of 12b80 was 157 days (range: 56–766). The cause of euthanasia was related to poor quality of life in five dogs, pathological fracture in four dogs, and septic shock in one dog.

### Post treatment bone tumor histopathology and necropsy examination

Tumor biopsies were repeated in five (50%) dogs at D57. Histopathology analysis of post treatment bone tumor biopsies revealed a stable mitotic index in two dogs: one dog treated at 8 mg/kg and one dog treated at 10 mg/kg. In addition, post treatment bone tumor biopsies were performed in nine (90%) dogs following euthanasia. An increased necrosis was noted in five dogs on post-mortem histopathology compared with the pretreatment histopathology analysis (dogs were treated with a cumulative dose of 12b80 of 4, 18, 20, 30 and 42 mg/kg) (Figure 2D–2I).

Three dogs had a full necropsy examination, including one dog treated with three cycles of 12b80 at dose 10 mg/kg, one dog treated with two cycles at 10 mg/kg that developed grade 5 septic shock and one dog treated with three cycles at 6 mg/kg and three additional cycles at 8 mg/kg. Two (2/3, 67%) dogs with metastasis identified on computed tomography at D57 had histopathology-confirmed metastatic disease at necropsy. Sites of metastasis included lungs, thoracic vertebrae, and ischium. Post-mortem examination of the dog with grade 5 septic shock revealed a duodenal ulceration and perforation. No evidence of cancer cells was noted at this site and no evidence of distant macroscopic metastatic disease was observed at necropsy. No gastrointestinal ulceration was reported on necropsy of the two other dogs.

## DISCUSSION

Spontaneous tumors in companion dogs represent a valuable model for human cancer biology and translational cancer therapeutics. The objectives of this dose escalation study were to evaluate the safety, tolerability and the MTD of intravenous 12b80 in client-owned dogs with naturally occurring osteosarcoma. Four doses were evaluated and the MTD of 12b80 in dogs was determined at 8 mg/kg, administered every three weeks for three cycles.

The results of this clinical trial showed that 12b80 was well tolerated in dogs. The safety profile of 12b80 included 31 TEAE with 94% (29/31) of grade 1 or 2 toxicities. The most frequently reported TEAE were IHR and gastrointestinal disorders. At the MTD of 8 mg/kg, all TEAE were reversible and no severe myelotoxicity was reported. Immediate hypersensitivity reactions were reported in four dogs treated with 12b80 and included signs of agitation, head shaking, pruritus, acute cutaneous erythema, or subcutaneous edema. All signs consistent with IHR were grade 1 and resolved with the reduction of the 12b80 infusion rate and prophylactic antihistamine medications. Immediate hypersensitivity reactions, caused by histamine release, have been reported in dogs in the first minutes of doxorubicin infusion [28]. Clinical signs usually resolve with drug withdrawal, and the majority of dogs tolerates resumption of the drug with a slower infusion rate. Others common TEAE with 12b80 were gastrointestinal disorders including vomiting, diarrhea and decreased appetite. All digestive disorders were mild to moderate (grade ≤ 2) and resolved spontaneously or with symptomatic medications. Here, no treatment discontinuation was noted due to IHR or gastrointestinal adverse events.

Severe TEAE occurred in two dogs treated at dose 10 mg/kg and were considered as DLT. One dog developed one grade 3 phlebitis with skin and subcutaneous necrosis which required a surgical intervention. Severe tissue ulceration and necrosis has been described with doxorubicin and bisphosphonate extravasation in dogs [29, 30]. Here, no injection site events were reported during the 12b80 administration, and no perivascular necrosis was observed. This dog had received the 12b80 IV through an indwelling catheter changed every 24 hours and this event occurred 7 days after the administration. The phlebitis and subcutaneous necrosis were suspected to be correlated with catheter indwell time following 12b80 administration. Following surgical debridement, the dog completely recovered, and no further complications were documented. Following this event, the protocol was adjusted using a catheter removed after each chemotherapy administration and no other injection site toxicity was reported. The second severe TEAE included one septic shock secondary to septic peritonitis, in a Rottweiler treated with two cycles of 12b80 at 10 mg/kg. This event occurred 14 days after the last dose of 12b80. Post-mortem examination revealed a duodenal ulceration and perforation and no evidence of cancer cells was noted at this site on histopathological examination. This dog had concomitant NSAID treatment initiated before enrollment. Long-term NSAID is one of the most common cause of gastroduodenal ulcer disease in dogs and particularly reported in Rottweilers [31]. It remains unclear whether this grade 5 TEAE was directly associated with 12b80 administration or related to the combination of concomitant NSAID medications allowed in the trial. Of interest, no gastrointestinal ulceration was reported on necropsy of the two other dogs included in the study and treated with a cumulative dose of 12b80 of 30 mg/kg and 42 mg/kg, respectively. These two dogs were also treated with long-term NSAID medications. Consequently, because two dogs experienced a DLT at dose 10 mg/kg, the dose escalation was discontinued and the MTD of 8 mg/kg was defined as the highest tolerated dose.

As a vectorized form of doxorubicin, the results of this canine trial demonstrate that 12b80 expends the MTD of doxorubicin up to four times the standard dose of 30 mg/m^2^ in dogs. Doxorubicin is a potent anthracycline antitumor antibiotic agent with cytotoxic effects and low therapeutic index. Dose dependent bone marrow suppression and cumulative cardiotoxicity limit its clinical usage in dogs [32]. In dogs, the MTD of doxorubicin has been determined at 30 mg/m^2^ associated with an increased risk of cardiotoxicity with cumulative doses greater than 240 mg/m^2^ [33]. In this trial, the dose of 12b80 was escalated from 4 mg/kg to 10 mg/kg, which represents an equivalent calculated dose of doxorubicin ranging from 48 mg/m^2^ to 159 mg/m^2^ per cycle and a cumulative calculated dose ranging from 48 mg/m^2^ to 477 mg/m^2^. One dog with intra-patient dose escalation of 12b80 received a cumulative dose of doxorubicin of 528 mg/m^2^. No statistically significant decrease in neutrophil counts was noted seven days post treatment. The serum biochemical evaluation profiles provided supportive evidence for the safety of 12b80. There was only one transient grade 2 increase in ALT and urea blood levels observed in one dog which spontaneously resolved before the following cycle. No hematological or biochemical adverse event requiring either dose reduction or treatment discontinuation was reported.

Repeated doses of doxorubicin can result in potentially life-threatening cardiotoxicity in dogs [28, 33]. Clinical findings of doxorubicin-induced cardiotoxicity include arrhythmias and decreased systolic function [34]. Histopathological findings of chronic irreversible cardiotoxicity include myofibrillary atrophy and degeneration, interstitial edema and fibrosis, swelling of the sarcoplasmic reticulum, and cytoplasmic vacuolation [32, 33]. In this trial, echocardiographic and electrocardiogram evaluation were repeated after completion of the three cycles of 12b80 in six (60%) dogs. No significant, sustained changes in cardiac parameters or cardiac function were identified in any dogs. Three (3/6, 50%) dogs had post-mortem cardiac histopathology performed. Those dogs received a cumulative dose of 12b80 of 20 mg/kg, 30 mg/kg and 42 mg/kg, which represents the equivalent calculated cumulative dose of doxorubicin of 264 mg/m^2^, 360 mg/m^2^ and 528 mg/m^2^, respectively. No cardiac DLT was observed on histopathology. Rare individual cell necrosis was noted in the dog treated at a 42 mg/kg cumulative dose and diffuse increased cardiomyocytes density was reported in the dog treated at a 20 mg/kg cumulative dose.

The results of this trial in companion dogs provide strong support that the vectorization of doxorubicin with an HBP significantly increases the MTD of doxorubicin in dogs, up to four times the standard dose of 30 mg/m^2^ without associated DLT. The bone targeting and local release of doxorubicin from the 12b80 in an acidic bone tumor environment is likely to have prevented excessive systemic interaction of the doxorubicin with normal proliferative cells and therefore limiting cellular damage due to oxidative reactions. Further pharmacokinetic analysis will be investigated in a phase II study comparing the plasma concentration of doxorubicin between 12b80 and nonvectorized doxorubicin.

Preliminary clinical antitumor activity of 12b80 was appreciable in the form of stable disease without evidence of metastatic disease in two dogs at D57. Quality of life monitoring showed stable quality of life to improved quality of life in the majority of the dogs treated with 12b80. Histopathology analysis of the bone tumor biopsies revealed a stable mitotic index in two dogs following three cycles of 12b80 and increased necrosis in five dogs on post-mortem analysis. The median survival time for dogs that completed the three cycles of 12b80 was 157 days (range: 56–766). For comparison, multiple reports described a survival ranging from 75 to 209 days for dogs with appendicular osteosarcoma treated with radiation therapy in combination with or without chemotherapy [20–22, 24].

In conclusion, this pilot dose escalation study demonstrates both a promising safety profile and preliminary clinical antitumor activity of 12b80 in a companion dog model of naturally occurring osteosarcoma. These results hold promise for the ongoing clinical development and potential translational relevance of 12b80 in dog and human patients with osteosarcoma, particularly for non-operable or metastatic bone disease. This pilot study will be followed by a phase II randomized double-blind study to compare the safety and antitumor activity of 12b80 and nonvectorized doxorubicin in client-owned dogs with naturally occurring osteosarcoma. This ongoing phase II study will explore pharmacokinetic/pharmacodynamic profiles and clinical efficacy with translational objectives for both dogs and humans.

## MATERIALS AND METHODS

### Dose escalation design and treatment

The study was conducted as a prospective, open-label, phase I dose escalation trial to assess the safety and tolerability, MTD and DLT of 12b80 in dogs with naturally occurring osteosarcoma. The study design and the data analysis were carried out by OCR (Oncovet-Clinical-Research), Parc Eurasanté, Loos, France. The clinical trial was conducted at Oncovet, Villeneuve d’Ascq, France. The 12b80 was provided by Atlanthera laboratory (Saint Herblain, France).

An accelerated dose-titration design, including one or two dogs enrolled at the initial doses was used followed by a 3 + 3 design [35]. Dogs started 12b80 treatment at a dose of 4 mg/kg (*n* = 1) and 6 mg/kg (*n* = 2). No DLT was observed at the initial doses and additional dogs were enrolled to determine the MTD in a 3 + 3 dose escalation design. In each cohort, a minimum of 3 dogs were included and received the same dose during the protocol. The next cohorts were treated at increasing dose levels according to the following criteria: (i) if no DLT was observed in the 3 dogs treated in a cohort, another three dogs could be treated at the next higher dose level; (ii) if one of the three dogs experienced a DLT, three more dogs were treated at the same dose level; (iii) if at least two dogs among a cohort of three or six dogs experienced a DLT, the dose escalation was discontinued. The recommended dose was defined as the dose level just below the dose level where two dogs developed DLT.

Four doses were evaluated: 4 mg/kg (cohort 1; *n* = 1), 6 mg/kg (cohort 2; *n* = 2), 8 mg/kg (cohort 3; *n* = 3), 10 mg/kg (cohort 4; *n* = 4) (Table 2). All dogs involved in the trial followed the same protocol over a period of nine weeks. The protocol included three cycles of 12b80 intravenous (IV) injections, administered every three weeks (day 1, day 22, day 43). For the doses 4 and 6 mg/kg, the total prescribed dose was divided in two administrations of 1-hour constant rate infusion over 24 hours. Immediate hypersensitivity reactions (IHR) were noted at the doses 8 and 10 mg/kg, thus the protocol was adjusted from two to three or four intravenous administrations of 1-hour of each over 48 hours and premedication with diphenhydramine (1 mg/kg, IV) and ranitidine (2 mg/kg, IV) were administered 20 minutes before the IV administration of the 12b80. The prescribed dose was diluted in sterile isotonic sodium chloride 0.9% solution to form a final concentration of 2 mg/ml. The drug was administered IV through an indwelling catheter placed into a cephalic vein using a syringe driver. The indwelling catheter was changed every 24 hours. One dog treated at the dose level 10 mg/kg developed one grade 3 phlebitis and necrosis at the IV injection site, thus the protocol was adjusted with a catheter removed after each chemotherapy administration. Dogs were hospitalized for three days during each cycle (days 1–3, days 22–24, and days 43–45).

### 12b80 (hydroxybisphosphonate linked doxorubicin)

**Figure 3:**
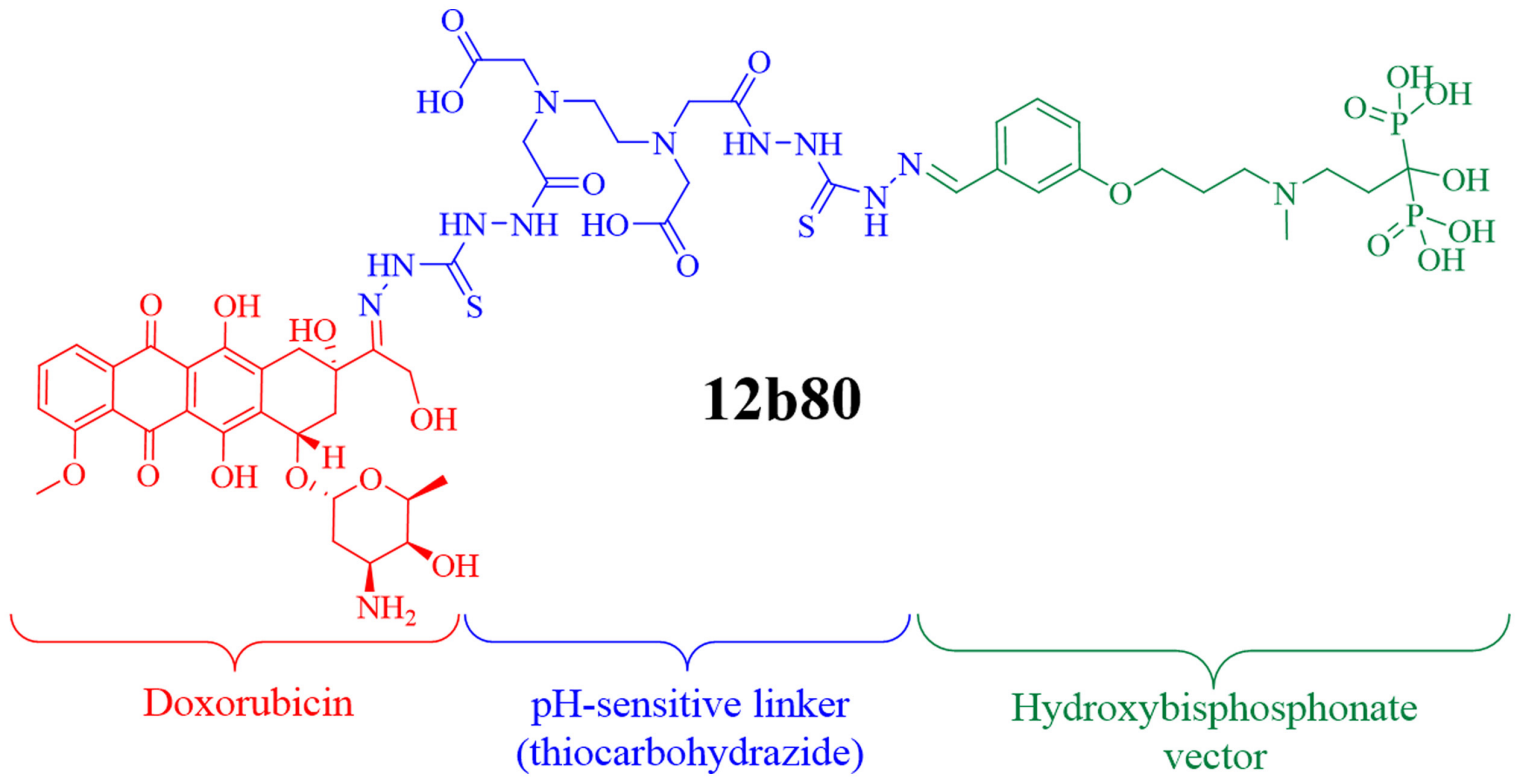
Chemical structure of 12b80. The molecule 12b80 combines doxorubicin bound to a bone targeting hydroxybisphosphonate vector using a pH-sensitive linker. This hydroxybisphosphonate linked doxorubicin compound was designed to specifically trigger doxorubicin release in an acidic bone tumor microenvironment.

The molecule 12b80 combines doxorubicin bound to a bone targeting HBP vector using a pH-sensitive linker (thiocarbohydrazide) (Figure 3). The design and synthesis of 12b80 are patented (WO 2016079327 A1). Each molecule of 12b80 contains one molecule of doxorubicin. The molecular weights of 12b80 and doxorubicin are 1431 g/mol and 543 g/mol, respectively. Toxicological data in rodents have shown that the MTD of 12b80 was more than three-fold higher than doxorubicin after a single injection and ten-fold higher after repeated doses of nine injections [25]. Toxicological data were preliminary evaluated in four Beagle dogs. Dogs were treated intravenously once a week for three weeks with intra-patient dose escalation of 12b80 between 3 and 16 mg/kg, resulting in cumulative doses ranging from 15 to 30 mg/kg. No death related to treatment was reported. Dose-dependent hematologic toxicity was observed with the highest dosage. Immediate hypersensitivity reactions were noted when the total dose was administered in a single injection but IHR were prevented during subsequent injections when the total prescribed dose was divided in two administrations. No DLT was observed on heart, bone and brain post-mortem histopathology.

In our trial, the first cohort of dogs was treated at a starting dose of 4 mg/kg (i.e., 1.5-fold higher than the equivalent calculated standard dose of doxorubicin in dogs). This dose was considered to be safe based on extrapolation from toxicological data profiles in rodents and Beagle dogs.

### Dog selection

Ten client-owned dogs presenting with naturally occurring osteosarcoma were enrolled in the trial. Dogs were considered eligible for inclusion if they were weighing 20–70 kg, diagnosed with untreated osteosarcoma, with or without evidence of macroscopic metastatic disease, with no evidence of significant biochemical abnormalities or blood cytopenia which precluded the use of cytotoxic drugs or bisphosphonates, and whose owners had declined standard anticancer therapies. Dogs were excluded from the study participation if they had surgery, radiation therapy, or had received prior systemic chemotherapy or bisphosphonates. Dogs with underlying cardiac disease were also excluded from the study population. Oral analgesics excluding bisphosphonates were deemed acceptable for the trial participation.

After enrollment, dogs were excluded from the trial in the instance of disease progression (including pathological fractures confirmed on imaging), DLT, or withdrawal based on the decision of the owner or investigator. Data collection was discontinued after the exclusion or death of the last dog included in the study.

Ethical approval was obtained from the OCR Ethical Committee before trial initiation. Dog owners were informed prior to the trial of available treatment options, including surgery with adjuvant chemotherapy and traditional palliative therapies. Written informed consent was obtained from the owner before enrollment of each dog. Owners were permitted to withdraw their dog from the trial at any time.

### Initial staging

At enrollment, all dogs had a complete clinical staging performed including a complete blood count, biochemistry panel, contrast-enhanced whole-body computed tomography, urine analysis, echocardiogram and electrocardiogram. Definitive diagnosis of osteosarcoma was confirmed based on histopathological assessment on tumor biopsy performed with 8-–11-gauge Jamshidi needles. Tumors were described as osteoblastic, chondroblastic, fibroblastic, and telangiectatic.

### Safety analysis

Safety analysis was conducted in all dogs that received at least one dose of 12b80. Safety analysis included treatment-emergent adverse events (TEAE), laboratory safety assessments and quality of life evaluation. Laboratory safety assessments included the measurement of a complete blood count and a chemistry profile (total proteins, albumin, globulins, urea, creatinine, glucose, ionized calcium, sodium, potassium, chloride concentrations and alkaline phosphatase, and alanine transaminase activities). Treatment-emergent adverse events were assessed during each scheduled visit and graded according to the VCOG-CTCAE, version 1.1 [26]. The adverse event grade assigned to each dog was based on the highest grade of hematological and biological toxicity reported and the highest grade of gastrointestinal or constitutional clinical signs noted. Dose-limiting toxicity was defined as any of the following TEAE: any grade 5 toxicity, any prolonged (*>* 48 hours) asymptomatic grade 4 neutropenia or thrombocytopenia, any grade ≥ 3 febrile neutropenia, and any grade ≥ 3 nonhematological toxicity.

Owners were questioned about signs of adverse clinical effects using a quality of life questionnaire adapted from Lynch and colleagues and completed at day 1 and day 57 [36]. A complete blood count was performed before and seven days after each cycle. Recovery of absolute neutrophils count to 1500 cells per μL and platelets count to 75000 platelets per μL was required before starting the next cycle. For dogs with prolonged (> 48 hours) asymptomatic grade 4 neutropenia, febrile neutropenia, and grade 3 gastrointestinal adverse events, the dose was reduced by 20% for the following cycles. If the neutrophil count was lower than 1500 /μL or the platelet count was lower than 75 000 /μL at the time of the next cycle, the chemotherapy administration was postponed for one week. If a delay of more than two weeks occurred, treatment was discontinued. Prophylactic broad-spectrum antibiotic therapy was administrated for dogs with grade ≥ 3 asymptomatic neutropenia. Dogs with febrile neutropenia, and grade ≥ 3 gastrointestinal adverse events were hospitalized and treated with supportive care and IV antibiotics. During drug administration, dogs were evaluated for hypersensitivity reactions. A physical examination was performed to identify any sign of agitation, head shaking, pruritus, acute cutaneous erythema, or subcutaneous edema. The heart rate, respiratory rate, and body temperature were monitored, and blood pressure was measured every 30 minutes during infusion. Cardiac evaluation including an echocardiogram and electrocardiogram was performed prior to treatment initiation and was repeated after completion of the three cycles of 12b80 (day 57).

### Assessment of response and follow-up

After completion of the three cycles (day 57), dogs underwent a complete end-staging including a complete blood count, biochemistry panel, contrast-enhanced whole-body computed tomography, urine analysis, echocardiogram and electrocardiogram. A bone tumor biopsy was repeated if no pathological fracture was identified on the computed tomography images. Response to treatment was defined according to the RECIST criteria v1.0 [27]. Follow-up contrast-enhanced whole-body computed tomography was performed four and six months after completion of treatment.

### Study endpoints

The primary endpoints of the study were safety, tolerability, DLT and the MTD. Safety analysis evaluated the occurrence of TEAE graded according to the VCOG-CTCAE v1.1 [26]. The secondary endpoints were the evaluation of the preliminary antitumor activity of 12b80 in dogs with osteosarcoma including quality of life assessment, overall response rate and overall survival evaluated by RECIST criteria v1.0 [27].

### Statistical analysis

Categorical variables were expressed as numbers and percentages. For continuous normally distributed variables, data were expressed as mean and standard deviation (SD), and groups were compared with *t*-test. For nonnormally distributed continuous variables, data were expressed as median and range, and groups were compared using Wilcoxon rank test. Overall survival (OS) was defined as the time between the first cycle initiation and death from any cause. Overall survival was used instead of disease specific survival since necropsy results were not available in all dogs to confirm osteosarcoma-related death. Results were considered statistically significant with a *P* value < 0.05. The statistical analysis was performed using SPSS version 24.0 software (SPSS A, Inc., Chicago, IL, USA).

## Abbreviations

ALP: alkaline phosphatase
ALT: alanine aminotransferase
DLT: dose-limiting toxicity
HBP: hydroxybisphosphonate
IHR: immediate hypersensitivity reactions
IV: intravenous
MTD: maximum tolerated dose
NSAID: nonsteroidal anti-inflammatory drugs
OS: overall survival
RECIST: response evaluation criteria in solid tumors
TEAE: treatment-emergent adverse events
VCOG: Veterinary Cooperative Oncology Group
